# Sidewalk Landscape Structure and Thermal Conditions for Child and Adult Pedestrians

**DOI:** 10.3390/ijerph15010148

**Published:** 2018-01-18

**Authors:** Young-Jae Kim, Chanam Lee, Jun-Hyun Kim

**Affiliations:** 1 Department of Forest Resources and Landscape Architecture, Yeungnam University, 280 Daehak-Ro, Gyeongsan, Gyeongbuk 38541, Korea; 2 Department of Landscape Architecture and Urban Planning, Texas A&M University, 3137 TAMU, College Station, TX 77843, USA; chanam@tamu.edu; 3 Landscape Architecture Program, School of Planning, Design and Construction, Michigan State University, 552 W Circle Drive, East Lansing, MI 48824, USA; junhkim@msu.edu

**Keywords:** street trees, grass areas, sidewalk vegetation, air temperature, walking, children

## Abstract

Walking is being promoted for health and transportation purposes across all climatic regions in the US and beyond. Despite this, an uncomfortable microclimate condition along sidewalks is one of the major deterrents of walking, and more empirical research is needed to determine the risks of heat exposure to pedestrians while walking. This study examined the effect of street trees and grass along sidewalks on air temperatures. A series of thermal images were taken at the average heights of adults and children in the US to objectively measure the air temperatures of 10 sidewalk segments in College Station, TX, USA. After controlling the other key physical environmental conditions, sidewalks with more trees or wider grass buffer areas had lower air temperatures than those with less vegetation. Children were exposed to higher temperatures due to the greater exposure or proximity to the pavement surface, which tends to have higher radiant heat. Multivariate regression analysis suggested that the configuration of trees and grass buffers along the sidewalks helped to promote pleasant thermal conditions and reduced the differences in ambient air temperatures measured at child and adult heights. This study suggests that street trees and vegetated ground help reduce the air temperatures, leading to more thermally comfortable environments for both child and adult pedestrians in warm climates. The thermal implications of street landscape require further attention by researchers and policy makers that are interested in promoting outdoor walking.

## 1. Introduction

Excessive heat exposure can cause many health problems, such as dehydration, heat strokes, and skin cancer [1,2,3]. In summer, heat exposure can increase heat stress and heat-related mortality rates [4,5,6]. Such heat-related risks are not equal across different population groups. For example, children suffer from greater exposure to the pavement surface, which has higher radiant heat, than adults because of their shorter height than adults [7,8]. Furthermore, children are often unaware or forgetful of the importance of sun protection [9].

An uncomfortable microclimate condition has also been reported to be one of the major deterrents of walking. Given the importance of walking as a popular source of obtaining healthy physical activity [10,11,12], and weather as a key barrier to walking, providing thermally comfortable pedestrian environments can bring many additional health benefits. Previous studies reported that undesirable thermal conditions for pedestrians can be mediated by street-level vegetation, such as trees [13,14,15,16], which can provide shaded surfaces, and grass cover, which can emit less long-wave radiation and lower the surface and air temperatures. On the other hand, the degree it can be mediated or the potential differences in the magnitude of mediation between adults and children are unclear.

Natural environments in neighborhoods such as trees and grass areas along sidewalks can help produce comfortable thermal conditions to encourage healthy outdoor activities among adults and children [17,18,19,20,21,22,23]. Several studies have found inverse relationships between the amounts of greenery in the neighborhood and the rates of childhood obesity [24,25,26,27]. In addition, green spaces in urban environments can relieve the mental stress among adults and children [23,28,29,30,31,32], and help to promote social interactions and the use of neighborhood public spaces [23,33,34,35]. Positive relationships between the presence of trees and well-maintained grass areas around buildings, and neighborhood safety (often manifesting as low rates of poverty, crimes, aggression, and violence) have also been reported [35,36,37]. Furthermore, the greater shade provided by street trees reduces the maintenance requirements of sidewalks, because it lowers pavement fatigue cracking, rutting, shoving, and other distresses to the surface [38].

Despite these benefits, empirical investigation into the measurable benefits of sidewalk vegetation on producing comfortable thermal conditions for outdoor activities has been limited. A few studies investigated the thermal environmental conditions, but most relied on simulation methods and focused on building designs or street canyons [39,40,41]. Regarding the overall green/vegetation structures as factors affecting thermal comfort, many studies have reported that such elements serve to cool the ambient air because tree cover/canopy blocks radiation and decreases the surface energy balance fluxes [13,42]. One study compared two courtyards that were designed with different materials and found that the courtyard planted with three mature trees and grass provided more comfortable thermal conditions than the other one with bare pavement and shading mesh [15]. On the other hand, only a few studies so far have used direct field measurements. The microclimate of streets, which are critical urban infrastructure functioning as both transportation facilities and physical activity resources, requires further attention. Streets hold particular significance because of their strong potential to support physical activity, which is demonstrated by the fact that streets are the most frequently used places for walking and outdoor physical activities [43].

To address some of the existing knowledge gaps in this area of research, the study has two main objectives. First, this study examined the effects of street trees and grass on the air temperatures along sidewalks, utilizing objective field measurements. Second, these effects were measured at the average height of children (outcome 1) and adults (outcome 2), and the differences between the adult and child measures (outcome 3) were examined. The findings from this study can offer insights into the potential for sidewalk landscape structures to help mitigate sidewalk air temperatures that child and adult pedestrians are exposed to while walking.

## 2. Methods

### 2.1. Study Setting and Data Measurement

This study was conducted in the City of College Station, TX, USA, which has a subtropical climate with 214 days a year above 26 °C [44]. Ten sidewalk segments (Types A to J) were selected based on having the same street orientation (North–South) and similar/adjacent locations, but different vegetation conditions (e.g., street trees and grass areas) along the sidewalk (Table 1). The selected sidewalks were paired to compare the air thermal conditions between the sidewalks with and without the green features (e.g., trees on one side, trees on both sides, grass on one side, and grass on both sides) in order to ensure comparable physical environmental conditions along the sidewalk segments other than the presence/amounts of green features.

**Figure 1:**
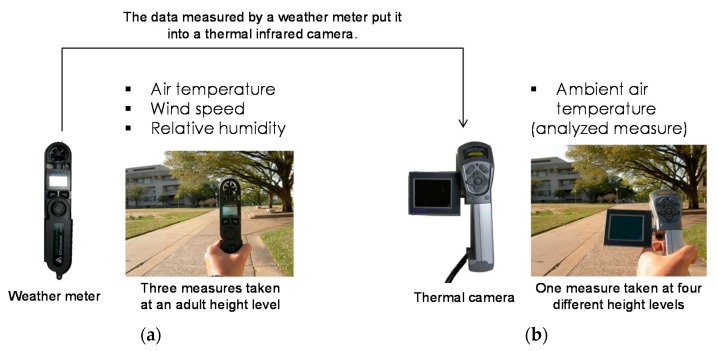
(**a**) Air temperature, wind speed, and relative humidity captured by a weather meter; and, (**b**) Ambient air temperature measured by a thermal infrared camera.

For each sidewalk segment, three measures, including air temperature, relative humidity, and wind speed were captured by a weather-meter (model: WM-350 WindMate, accuracy: air temperature ±2 °C/wind ±3% when aligned with winds axis/relative humidity ±3%, range: air temperature −20 °C to 70 °C/wind 0.8 mph to 89 mph/relative humidity 5–95%) [45] and these measures were entered into a thermal infrared camera to capture thermal images and estimate the ambient air temperatures from the thermal images (model: SATIR E8-N, accuracy: ±2 °C, temperature range: −20 °C to 250 °C) [46] (Figure 1). The ambient air temperatures measured by the thermal camera were used as outcome measures in this study. Figure 1 shows the data collection instruments and protocols.

**Figure 2:**
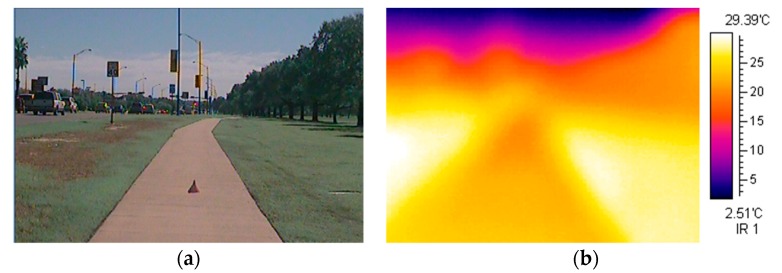
(**a**) Sidewalk image taken 25 feet away from the orange traffic cone used as a distance and orientation guide; and, (**b**) Thermal image taken at an adult height level.

Figure 2 gives an example of a sidewalk photograph image and its thermal image captured by the thermal infrared camera used in this study. Four thermal images were taken each time at four different heights, representing the average heights of children (121.5 cm for a 7-year-old, 138.4 cm for a 10-year-old) and adults (163.3 cm for female adults and 177 cm for male adults) [47]. The child height measures may also be relevant to people using a wheel chair or a stroller. The thermal images were taken 25 feet (ten steps) away from a measuring point indicated with an orange traffic cone used as a distance and orientation guide to ensure consistent sight lines and distances across all of the thermal images (Figure 2a). Sidewalk width and grass width were measured with a tape measure. To determine the tree height and canopy width, a photo analysis using a human figure was used as a height reference. A researcher who is 175 cm tall stood next to the street trees, while holding a 175-cm long scale bar perpendicular to the trees. Each photograph was taken from each sidewalk segment selected. Based on the photo taken from each street tree, the tree height and canopy width on the sidewalks were estimated as an average of all the trees along the segment. Thermal images and field measurements for each sidewalk segment were taken three times a day (10 a.m., 2 p.m. and 6 p.m.) on six sunny days from June to September in 2010. Because of the proximate/adjacent locations of all sidewalk segments, the travel times between the segments were less than 5 min and no major changes were recorded among the measures from the same time window in terms of the traffic and other non-physical conditions. The average air temperature, relative humidity, and wind speed for the six days was 32.8 °C (range: 26.8 °C~39.3 °C), 55.7% (37.8~85.2%), and 2.7 km/h (0 km/h~8.8 km/h), respectively. Our study protocol was designed to ensure consistency and minimize the influence of other confounding factors.

### 2.2. Data Analysis

After conducting descriptive statistics analysis, a series of paired *t*-tests were performed to analyze the mean differences in air temperatures between the two sidewalk settings in each pair. To predict the combined effects of street trees and grass on the air temperatures along sidewalks, ordinary least squares (OLS) regressions were conducted, while considering the 0.01, 0.05, and 0.10 levels of significance for interpreting results [48].

Three dependent variables were used for multivariate analyses: (1) air temperatures measured at the children’s height (average between 7 and 10-year-old children); (2) air temperatures measured at the adults’ height (average between male and female adults); and, (3) air temperature differences between the child and adult measures. Six paired interaction terms (independent variables) between street trees (categorical variable, 0: no tree, 1: tree on one side, 2: tree on both sides) and grass areas (categorical variable, 0: no grass, 1: grass on one side, 2: grass on both sides) were analyzed to predict their combined impacts on each of the three outcome variables. The interaction terms were comprised of six dummy variables; (1) Tree0*Grass0 (1 if no tree and no grass; 0 otherwise); (2) Tree0*Grass1 (1 if no tree and grass on one side); (3) Tree0*Grass2 (1 if no tree and grass on both sides); (4) Tree1*Grass1 (1 if tree on one side and grass on one side); (5) Tree1*Grass2 (1 if tree on one side and grass on both sides); and, (6) Tree2*Grass2 (1 if tree on both sides and grass on both sides). By using Tree0*Grass0 (no tree and no grass) as the reference group and the other five dummy variables as independent variables, the OLS regression estimated the effect of each independent variable on the three outcome variables.

## 3. Results

### 3.1. Independent Effect of Street Trees and Grass on Air Temperature

Table 2 lists the *t*-test results comparing the mean temperature difference between the two sidewalk segments in each pair, which shows the independent effect of street trees and grass on the air temperature. Four paired groups with different numbers of street trees were compared to assess the influence of existing trees on the mean air temperature, while controlling for other physical conditions, such as sidewalk width and the presence of grass. When only the afternoon mean temperatures were used, the mean differences were significant at the 0.01 level in all four pairs. On the other hand, only two out of the four pairs were significant when all of the temperature measures (taken at 10 a.m., 2 p.m. and 6 p.m.) were used. The mean temperature of the type A sidewalk (no tree) was 1.03 °C higher than that of type C (trees on one side) based on the afternoon mean temperature. The type D (no tree) sidewalk measured in the afternoon was 1.44 °C higher than the type F (trees on one side) sidewalk. The mean air temperature of type A (no tree) in the afternoon was 2.01 °C higher than type E (trees on one side), and type C (trees on one side) was 0.99 °C higher in the afternoon than type E (tree on both sides). The overall results from the paired analyses assessing the impacts of street trees indicated that having more trees along sidewalks decreased the ambient air temperatures, particularly in the afternoon hours.

Four sidewalk pairs selected to test the roles of grass buffer along sidewalks showed statistically significant differences at the 0.01 level. A 4.20 °C mean temperature difference was observed in the afternoon between type B (no grass) and type A (grass on both sides), and a 5.80 °C mean temperature difference was noted in the afternoon between type D (grass on one side) and type A (grass on both sides). The mean of air temperature of type F (grass on one side) in the afternoon was 5.29 °C higher than that of type C (grass on both sides), and type H (grass on one side) was 1.01 °C higher in the afternoon than that of type G (grass on both sides). These findings suggested that grass areas along sidewalks played a significant role in decreasing the ambient air temperature, and the temperature reduction was greater in the afternoon, with the exception of one pair (types H and G).

### 3.2. Combined Effect of Street Trees and Grass on Air Temperature

Table 3 presents the results of the combined effects of street trees and grass areas on the air temperature. Two regression models, one using the air temperature for children and the other using the air temperature for adults, were estimated. The results showed similar outcomes in that compared to “no trees and no grass (Tree0*Grass0)”, “no trees and grass on both sides (Tree0*Grass2)”, “trees on one side and grass on both sides (Tree1*Grass2)”, and “trees on both sides and grass on both sides (Tree2*Grass2)” were significantly associated with lower air temperatures by up to 3.589 °C, 3.115 °C, and 3.667 °C at the child height, respectively; and, 3.589 °C, 3.115 °C, and 3.667 °C at the adult height, respectively.

These findings also showed that children are exposed to higher air temperature than adults. For example, the mean air temperature measured at the child height in the afternoon was 38.65 °C (38.85 °C for 7-year-old and 38.45 °C for 10-year-old), which was 1.33 °C higher than that measured at adult heights (37.75 °C for female adults 36.89 °C for male adults). To further examine whether trees and grass cover along sidewalks help reduce the air temperature differences between children and adults, six paired interaction terms between street trees and grass strips were examined further to predict the mean child-adult temperature difference variable (Model 3 in Table 3). The Tree1*Grass2 variable was moderately significant (*p* = 0.050), indicating that the sidewalk with trees on one side and grass on both sides had a 0.613 °C lower air temperature gap between children and adults, compared to the sidewalk with no tree and no grass. The coefficient of Tree1*Grass2 variable was –0.557 (*p* = 0.093), meaning that the sidewalk with trees and grass on both sides reduced the air temperature difference between children and adults by 0.557 °C, when compared to the sidewalk with no tree and no grass. In addition to reducing the overall air temperature, trees, and grass cover along sidewalks have been shown to reduce the temperature gap between children and adults.

## 4. Discussion

This study examined the cooling effects of sidewalk vegetation on the thermal conditions for child and adult pedestrians. This is one of the few studies to examine the relationship between sidewalk vegetation conditions and ambient air temperatures, using objectively measured data captured from the real-world environments.

The independent effects of trees on thermal conditions from the paired *t*-tests suggested that planting more trees along the sidewalks could decrease the air temperature. On the other hand, the independent effect of trees varies according to the presence of grass areas along the sidewalks. In a comparison between “no trees” and “trees on one side”, the difference in air temperature was lower in type A (no tree) and type C (tree on one side), both of which had grass areas on both sides (1.03 °C), when compared to type D (no tree) and type F (tree on one side), both of which had grass on only one side (1.44 °C). Furthermore, type E (trees and grass on both sides) showed a greater temperature difference when compared to type A (no trees but with grass on both sides) (2.01 °C), versus type C (trees on one side but with grass on both sides) (0.99 °C). These results are consistent with previous studies that reported the cooling effect of vegetation under simulated or experimental conditions [13,14,15,40].

The independent effects of grass areas on the air temperature showed that the presence of grass areas along the sidewalks plays a significant role in reducing the ambient air temperature. On the other hand, type A (grass on both sides) showed a greater air temperature difference than type D (grass on one side) (5.8 °C), as compared with type B (no grass) (4.2 °C). The reason for this counter-intuitive result might be associated with the condition of the type D sidewalk. Type D had very narrow grass areas (0.65 m), which was built with a brick wall on the side that could absorb and store heat. This condition may radiate energy from the asphalt pavement, which could help to increase the heat on the brick wall due the close distance between the wall and the sidewalk segment. In addition, there was concrete-paved parking behind the narrow grass that keeps a large amount of solar radiation reflected from its surface. These special conditions appear to explain this result.

Grass analysis also highlighted the importance of grass locations as a buffer between sidewalks and car roads. When compared to the sidewalk having grass on both sides, sidewalks with grass areas on one side only between the sidewalks and roads showed higher ambient air temperatures, as expected. On the other hand, the difference in temperature was smaller when the grass was located outside abutting the vehicular lanes as compared to the grass located inside toward the building side (type H versus F with difference of 1.01 °C versus 5.29 °C, respectively). This result implies that grass or other vegetated ground covers in the buffer area between the vehicular roadways and sidewalks may be more important for mitigating the ambient air temperatures.

The combined effects of trees and grass on the thermal conditions were examined by multivariate analyses using the six interaction terms, depending on the physical conditions regarding the presence of trees and grass. While the child and adult regression models showed similar results, the coefficients of the significant interaction terms from the child model (Model 1) were greater than those from the adult model (Model 2). This suggests that both planting trees and adding grass strips along sidewalks has a stronger impact on decreasing the ambient air temperature with greater benefits to child pedestrians. Furthermore, planting trees and grass on either one side or both sides of the sidewalks also decrease the difference in air temperatures measured at both the child and adult heights. In terms of the air temperature difference between children and adults, only one interaction term, Tree1*Grass2 (trees on one side and grass on both sides), remained significant (Model 3). For a more rigorous examination of these combined effects of trees and grass, future research should better control the types of tree species, tree heights, tree canopy sizes, and grass widths.

Like most empirical studies on this topic, this study also had several limitations and the results need to be interpreted within its limited scope and context. First, as an exploratory pilot study, the findings from this study are not generalizable to other areas with different climatic conditions and different street design characteristics. Second, there is a potential for a non-randomized bias because sidewalk segment measures were taken repeatedly on six different sunny days; hence, the results of the paired *t*-tests may be biased to some extent. Third, the thermal images taken by the infrared thermal camera that was used for this study embrace several elements of the scenes up to the sky, which are less associated with the surface conditions of the pavements. On the other hand, this study measured the sidewalk segments at four different heights, and the same portions of the sky in each thermal image were obtained with the camera. Fourth, as a cross sectional field study, it was not possible to completely control other potential confounding factors in the selected built environmental conditions (e.g., a brick wall, proximity to vehicle roads from sidewalks, distance between trees, and tree species), and not feasible to find a completely matching pair. Therefore, this study carefully selected the sidewalk segments to maximize the comparability by ensuring similarity in other non-test conditions within each pair, and to minimize the influence of other spatial factors by ensuring their proximate/adjacent locations and minimal time gaps among the measurements within each time window. Future research with stronger experimental designs using simulated sidewalk conditions can better handle the other potential confounders and increase the ability to detect more accurately the temperature mitigating impact of different street types/designs, spacing between trees, and tree species. Furthermore, future research will need to consider the effects of the surface albedo on the microclimate conditions by testing different types of sidewalk materials.

This study makes several contributions to the literature on this topic. First, in contrast to previous studies on street or sidewalk thermal conditions, which relied mainly on simulated building arrangements [40,41], street canyons [39], and outdoor experimental setup [15], this study measured the thermal conditions of actual sidewalk segments that people use most frequently as part of their daily routine. Second, multivariate analyses were performed to help detect more statistically meaningful results. Third, similar to the findings from a previous study, which showed a greater improvement of thermal comfort provided by a combination of shade trees and grass when compared to the effect from a single landscape treatment [15], this study showed a greater cooling effect when both trees and grass were installed along the sidewalk. In addition, the potential mitigation effect of sidewalk landscape structure on air temperature was identified at both the child and adult height levels, which suggests that there is no need to design different landscape structures to support the thermal comport of adults versus children. On the other hand, the configuration of street trees and grass along sidewalks may reduce the difference in ambient air temperatures that the children versus adults are exposed to. To provide more comfortable thermal conditions for children who tend to be more vulnerable to heat-related risks, planting both understory vegetation and canopy trees appears important. Future research may extend beyond the scope of this study by examining the relationship between the actual thermal comfort experienced by child and adult pedestrians and the sidewalk vegetation conditions. Fourth, for sidewalk buffers that are not wide enough to install both canopy trees and grass areas, the findings suggest that a grass strip buffer located between vehicle roads and sidewalks are appropriate for producing pleasant microclimate for both children and adults as it controls the heat that is generated by both vehicles and asphalt pavement of the roadway. In addition to the air temperature-alleviating potential of grass that this study explored, other types of understory vegetation can also help to improve the thermal conditions of sidewalks. Future studies should examine the potential and varying effects of different types (different sizes/heights/species) of understory vegetation on the air temperatures of sidewalks. Finally, afternoon measures showed a much greater air temperature reduction from trees and grass. A previous study found that the cooling effect of plants is well detected from the afternoon to sunrise of the next day [49]. Another study utilizing satellite images to assess the cooling effect of vegetated areas on the land surface temperature revealed such an effect to be stronger in the afternoon [50]. This study also showed that afternoon measures might be more sensitive and effective in detecting the temperature reduction function of sidewalk vegetation.

## 5. Conclusions

The presence of trees and grass areas along the sidewalks can help reduce the air temperatures for both child and adult pedestrians, not only by providing shade, but also by limiting the amount of solar radiation reflected from sidewalk surfaces. Furthermore, the sidewalk vegetation can help mitigate the air temperature difference between children and adults. Therefore, policy makers, planners, and designers should consider the thermal implications of street designs for health and transportation purposes. The thermal benefits of planting more trees and grass strips along sidewalks should be considered better during the relevant decision-making process.

## Figures and Tables

**Table 1:** Characteristics of the sidewalk segments selected for this study (Types A to J) ^†^.

	Type A	Type B	Type C	Type D	Type E
Photo	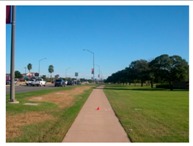	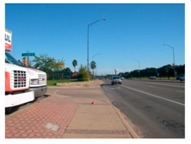	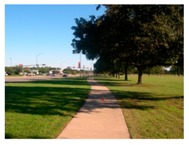	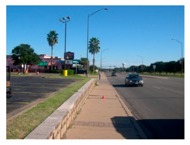	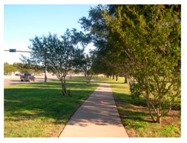
• Sidewalk width	1.83 m	1.76 m	1.82 m	1.85 m	1.83 m
• Presence of tree	No	No	Yes one side	No	Yes on both sides
• Tree height	-	-	9.02 m	-	6.91 m
• Canopy width	-	-	15.01 m	-	9.44 m
• Presence of grass	Yes on both sides	No	Yes on both sides	Yes on one side	Yes on both sides
• Grass width	4.61 m	-	7.40 m	1.01 m	3.16 m
	**Type F**	**Type G**	**Type H**	**Type I**	**Type J**
Photo	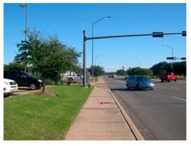	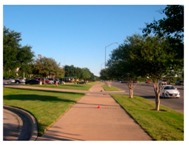	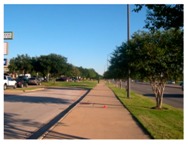	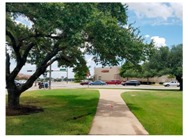	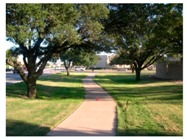
• Sidewalk width	1.85 m	3.05 m	3.05 m	1.83 m	1.83 m
• Presence of tree	Yes on one side	Yes on one side	Yes on one side	Yes on one side	Yes on both sides
• Tree height	4.52 m	5.13 m	5.13 m	10.62 m	9.87 m
• Canopy width	4.80 m	6.15 m	6.15 m	16.33 m	12.48 m
• Presence of grass	Yes on one side	Yes on both sides	Yes on one side	Yes on both sides	Yes on both sides
• Grass width	3.51 m	2.95 m	3.35 m	4.68 m	4.68 m

Note: The heights and widths of the trees and grass are the mean values. ^†^ Field measurements and thermal images for each sidewalk segment were taken three times a day on six sunny days.

**Table 2:** Paired comparison of the independent effects of street trees and grass on the air temperature.

Categories	Paired Sidewalk Types	Entire Day (10 a.m., 2 p.m., 6 p.m.)				Afternoon (2 p.m., 6 p.m.)			
		N ^†^	Mean (°C)	SD	Diff. (°C)	N ^‡^	Mean (°C)	SD	Diff. (°C)
Street Trees	Type D (No tree w/grass on one side)	68	38.62	4.89	0.83 *	44	41.80	2.46	1.44 **
	Type F (Tree on one side w/grass on one side)	68	37.79	4.42		44	40.36	2.80	
	Type A (No tree w/grass on both sides)	72	34.73	3.69	0.29	48	36.66	2.57	1.03 **
	Type C (Tree on one side w/grass on both sides)	72	34.44	2.96		48	35.63	2.57	
	Type A (No tree w/grass on both sides)	72	34.73	3.69	0.97 **	48	36.66	2.57	2.01 **
	Type E (Tree on both sides w/grass on both sides)	72	33.76	2.81		48	34.65	2.76	
	Type C (Tree on one side w/grass on both sides)	72	34.44	2.96	0.68 **	48	35.63	2.57	0.99 **
	Type E (Tree on both sides w/grass on both sides)	72	33.76	2.81		48	34.65	2.76	
Street Grass	Type B (No grass w/o trees)	72	38.03	5.02	3.30 **	48	40.86	3.27	4.20 **
	Type A (Grass on both sides w/o trees)	72	34.73	3.69		48	36.66	2.57	
	Type D (Grass on one side w/o trees)	72	39.24	5.40	4.51 **	48	42.46	3.23	5.80 **
	Type A (Grass on both sides w/o trees)	72	34.73	3.69		48	36.66	2.57	
	Type F (Grass on one side w/trees on one side)	68	37.79	4.42	3.79 **	44	40.36	2.80	5.29 **
	Type C (Grass on both sides w/trees on one side)	68	34.01	2.42		44	35.08	1.84	
	Type H (Grass on one side w/trees on one side)	72	36.63	3.17	1.06 **	48	37.69	3.24	1.01 **
	Type G (Grass on both sides w/trees on one side)	72	35.58	3.23		48	36.67	3.18	

** *p* < 0.01; * *p* < 0.05. Note: Diff. indicates a temperature difference between the paired sidewalks. SD. stands for standard deviation. ^†^ indicates the number of observations measured in the morning (10 a.m.) and the afternoon (2 p.m. and 6 p.m.) on six sunny days (4 heights * 3 times * 6 days = 72). ^‡^ indicates the number of observations measured in the afternoon (2 p.m. and 6 p.m.) on six sunny days (4 heights * 2 times * 6 days = 48).

**Table 3:** Parameter estimates of the combined effects of street trees and grass along sidewalks on thermal air temperatures measured at child and adult heights and temperature in difference between child and adult measures, using the interaction terms in regression models.

Interaction Terms	Child Temp. (Model 1)		Adult Temp. (Model 2)		Diff. in Temp. (Child–Adult) (Model 3)	
	Coef.	*p* > |*t*|	Coef.	*p* > |*t*|	Coef.	*p* > |*t*|
Tree0*Grass0 (no trees & no grass)	Ref.		Ref.		Ref.	
Tree0*Grass1 (no trees & grass on one side)	1.172	0.361	1.236	0.335	−0.064	0.867
Tree0*Grass2 (no trees & grass on both sides)	−3.589	0.006	−3.019	0.019	−0.569	0.136
Tree1*Grass1 (trees on one side & grass on one side)	−0.569	0.608	−0.554	0.617	−0.015	0.963
Tree1*Grass2 (trees on one side & grass on both sides)	−3.115	0.003	−2.502	0.018	−0.613	0.050
Tree2*Grass2 (trees on both sides & grass on both sides)	−3.667	0.001	−3.113	0.006	−0.557	0.093
Number of observation Adjust R-squared	N ^†^ = 180 R^2^ = 0.150		N ^†^ = 180 R^2^ = 0.108		N ^†^ = 180 R^2^ = 0.031	

Note: Coef., Diff., and Temp. indicate coefficient, difference, and temperature, respectively. ^†^ indicates the number of observations measured in the morning (10 a.m.) and afternoon (2 p.m. and 6 p.m.) on six sunny days (10 sidewalks * 3 times * 6 days = 180).
